# Bacterial cellulose-derived carbon nanofibers as both anode and cathode for hybrid sodium ion capacitor†

**DOI:** 10.1039/c9ra10225f

**Published:** 2020-02-24

**Authors:** Jiaxin Xu, Zhanying Liu, Fang Zhang, Jie Tao, Laifa Shen, Xiaogang Zhang

**Affiliations:** Jiangsu Key Laboratory of Electrochemistry Energy Storage Technologies, College of Material Science and Engineering, Nanjing University of Aeronautics and Astronautics Nanjing 210016 People's Republic of China zhangfang@nuaa.edu.cn

## Abstract

Hybrid ion capacitors (HICs) based on insertion reactions have attracted considerable attention due to their energy density being much higher than that of the electrical double-layer capacitors (EDLCs). However, the development of hybrid ion capacitors with high energy density at high power density is a big challenge due to the mismatch of charge storage capacities and electrode kinetics between the battery-type anode and capacitor-type cathode. In this work, N and O dual doped carbon nanofibers (N,O-CNFs) were combined with carbon nanotubes (CNTs) to compose a complex carbon anode. N,O dual doping effectively tuned the functional group and surface activity of the CNFs while the integration of CNTs increased the extent of graphitization and electrical conductivity. The carbon cathode with high specific surface area and high capacity was obtained by the activation of CNFs (A-CNFs). Finally, a hybrid sodium ion capacitor was constructed by the double carbon electrode, which showed a superior electrochemical capacitive performance. The as-assembled HIC device delivers a maximum energy density of 59.2 W h kg^−1^ at a power density of 275 W kg^−1^, with a high energy density of 38.7 W h kg^−1^ at a power density of 5500 W kg^−1^.

## Abstract 2

A hybrid sodium ion capacitor is constructed by the double carbon electrode, whose precursors are both from nanofibers of bacterial cellulose, showing a superior electrochemical capacitive performance.

## Graphical abstract

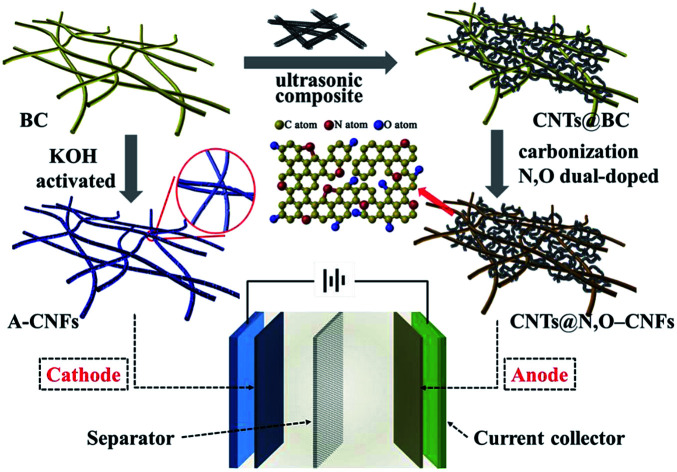

## 1. Introduction

As a new-type of energy storage device, non-aqueous hybrid ion capacitors (HICs) based on intercalation reaction have attracted considerable attention due to their energy density that is much higher than that of electrical double-layer capacitors (EDLCs).^1–4^ Generally, HICs are defined as the devices where a battery-type electrode and a capacitive-type electrode are combined with organic Li/Na salt-containing electrolyte.^5^ The battery-type electrode worked on an intercalation/deintercalation reaction to provide high energy, while the capacitive-type electrode is based on a physical adsorption/desorption process to offer high power.^6,7^ The combination of intercalation-type anode and adsorption-type cathode has opened a new way to obtain energy storage devices with both high energy and power density, as well as long cycling life.^8^ However, the mismatch of kinetics between the two electrodes is an issue, making the high energy density of HICs achievable only when the power density is low.^9,10^ In addition, the discrepancy in the specific capacities between the anode and cathode need to be balanced as well.^6^ Similar to supercapacitors, HICs are supposed to have long cycling life, such as of about 50 000 cycles or even more, so the coulombic efficiency (CE) of HICs is crucial. Furthermore, the Li or Na content in HICs is more limited compared to conventional Li/Na-ion batteries, making the solid-state electrolyte interphase (SEI)-induced CE loss very intractable. Thus, the prelithium/sodium technology becomes more and more important for HICs to compensate the Li or Na loss.

Electrode materials play a crucial role in determining the performance of HICs. To develop suitable electrode materials with large specific capacities and reaction kinetics that can fully utilize the hybrid systems is still a big challenge. It is necessary to explore a battery-type anode with fast kinetics to maintain fast response against a capacitive-type cathode, so that the kinetic progress between the two electrodes can be matched. Besides, the specific capacity and working potential of the cathode are expected to be further improved to enhance the energy density of HICs. Due to porous carbon that can facilitate Li^+^ or Na^+^ diffusion in the solid phase and increase the contact areas between the electrode and the electrolyte,^11–13^ a carbon nanomaterial with suitable porosity and specific surface area is the best option to boost the reaction kinetics of the battery-type negative electrode. In addition, hetero-atom doping has been demonstrated as an effective way to regulate the microstructure of carbon materials and thus, the electrochemical storage performance of carbon materials can be tuned.^14–16^ Our previous work has demonstrated that N,P and N,O dual-doped carbon nanomaterials showed significantly enhanced Na storage performance.^17,18^ Due to the working principle of cathode materials in HICs that is the same as that of EDLCs, the main factors that affect the capacitance performance are the specific surface and pore size distribution of the carbon materials. The high specific surface area and suitable pore size distribution are advantageous for achieving high specific capacitance and excellent rate performance.^19^

Carbon nanofiber (CNFs) aerogels, which are composed of interconnected carbon nanofibers, are of considerable interest due to their unique physical and chemical properties. Carbon nanofiber aerogels obtained by the pyrolysis of bacterial cellulose (BC) consist of ultrafine carbon nanofibers, which naturally have a porous network structure; thus, they are good choices for electrode materials of HICs.^20^ Unlike graphene and carbon nanotubes (CNTs), BC-derived carbon nanofibers are intrinsically hard carbon, which possesses excellent Na storage performance.^20^ In view of the above advantages, Na-ion HICs based on BC-derived carbon nanofibers are expected to be constructed to improve the electrochemical performance of HICs.

**Scheme 1:**
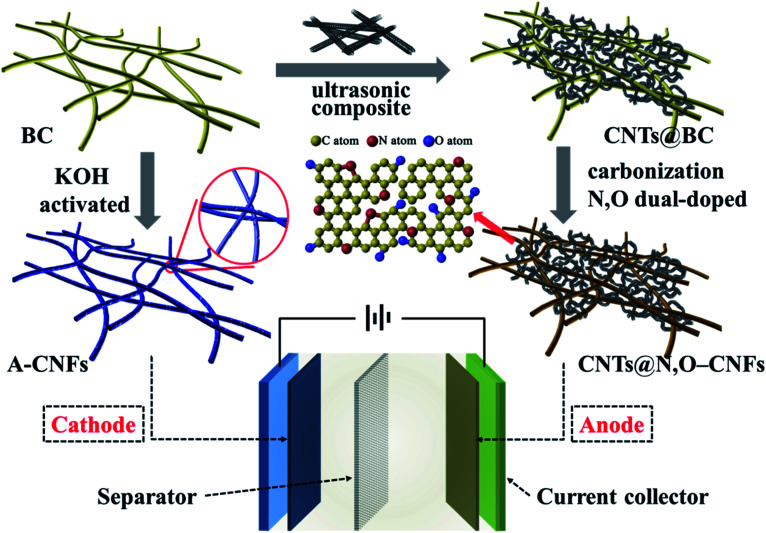
Schematic diagram of the synthesis and assembly of HIC.

Considering the fundamental difference in anode and cathode materials for HICs, in this work, we tailored the synthetic process to develop carbon nanofiber-based electrode materials for HICs using BC hydrogel as the precursor. To be specific, a facile and scalable methodology was developed to synthesis N and O double-doped CNFs (N,O-CNFs)/CNTs composite (CNTs@N,O-CNFs) that was used as the negative electrode of HICs. It was demonstrated that the combination of CNTs and N,O double doping effectively increased the active sites and reduced the charge transfer resistance, which improved the electrode kinetics of the carbon nanofibers. In contrast, the positive electrode was fabricated *via* chemical activation by KOH that provided abundant nanopores and a large specific surface area; thus, the electrode exhibited high specific capacity and ideal double-layer capacitive performance. Consequently, a sodium ion HIC device assembled by BC-derived double carbon electrode revealed excellent energy density and power density with long cycle life (Scheme 1).

## 2. Experimental section

### 2.1 Materials' preparation

#### Synthesis of CNTs@N,O-CNFs

Multi-walled carbon nanotubes (MWCNTs) and BC hydrogel were firstly integrated in an aqueous solution by ultrasonic dispersion, then the hydrogel was treated *via* a freeze drying method to obtain the composite CNTs@BC. Then, the composite was carbonized in a tube furnace at 1000 °C for 2 h under a nitrogen atmosphere at a heating rate of 2 °C min^−1^. Subsequently, the N and O doping process of the carbonized product (named as C-CNTs@CNFs) was performed. Specifically, C-CNTs@CNFs was dissolved in a mixed solution of HNO_3_ and H_2_SO_4_ with a volume ratio of 3 : 1 under stirring at 60 °C for 6 h. The precipitate was washed with deionized water several times and dried in an oven at 60 °C overnight. Finally, the dried product was put in 30 mL ethylenediamine solution and stirred at 70 °C for 6 h. The final product was thoroughly washed with deionized water and dried at 60 °C; this product was named as CNTs@N,O-CNFs.

#### Synthesis of A-CNFs

The BC hydrogel was firstly freeze-dried to obtain the BC aerogel and the obtained product was heated to 1000 °C in a tube furnace at a heating rate of 2 °C min^−1^ in a nitrogen atmosphere and was maintained at this temperature for 2 h. Subsequently, a certain amount of the carbonized products (named as C-CNFs) and KOH were mixed in the mass ratio of 1 : 2 and 1 : 4, respectively, and then transferred to a tube furnace; the carbonization parameters were set as following: an activation temperature of 800 °C with a heating rate of 5 °C min^−1^ for 2 h. The activated products (named as A-CNFs) in the mass ratio of 1 : 2 and 1 : 4 were named as A-CNFs-2 and A-CNFs-4, respectively.

### 2.2 Materials' characterization

The XRD patterns of CNTs@N,O-CNFs and A-CNFs were obtained by the X-ray diffraction (XRD) method using a Bruker D8 Advance powder X-ray diffractometer with Cu Kα radiation (*λ* = 0.154 nm). The surface elemental composition of the CNFs was identified by photoelectron spectroscopy (XPS) surface analysis (ESCALAB, 250xi, ThermoFisher). The morphologies of the carbon materials were examined by a field emission scanning electron microscope (FESEM, JSM-7800F, Japan) and a transmission electron microscope (TEM, Tecnai 12, Holland). The energy dispersive analysis (EDS) was performed using FESEM. The nitrogen adsorption/desorption isotherms were tested at 77 K on an adsorption/desorption apparatus (Quantachrome, NOVA2000e, USA). The specific surface area was determined by the Brunauer–Emmett–Teller (BET) method and the pore size distribution was calculated by the non-local density functional theory (NLDFT).

### 2.3 Electrochemical measurements

Electrochemical measurements are carried out *via* CR2032-type coin cells in a glove box using Na metal as the counter electrode. The slurry of the electrodes for electrochemical testing was prepared by mixing the electroactive material, acetylene black, and carboxymethyl cellulose in the mass ratio of 8 : 1 : 1, which was coated on current collectors (Cu foil for anode and Al foil for cathode) and dried at 60 °C overnight. The electrolyte was 1 M NaClO_4_ in ethylene carbonate (EC) : propylene carbonate (PC) (1 : 1 in volume) with 5 wt% fluoroethylene carbonate (FEC) added to it. The mass of both CNTs@N,O-CNFs and A-CNFs was 0.8 mg in the half cells but in the full HICs device, the mass of the anode and cathode was 0.8 mg and 1.6 mg, respectively. Cyclic voltammetry (CV) and electrochemical impedance spectroscopy (EIS) tests were performed on the CHI750D electrochemical workstation. Galvanostatic charge–discharge and long-term cycle performance tests were conducted on a Land CT2001A battery testing system. All the electrochemical measurements were conducted at room temperature.

## 3. Results and discussion

### 3.1 Negative electrode materials

**Fig. 1:**
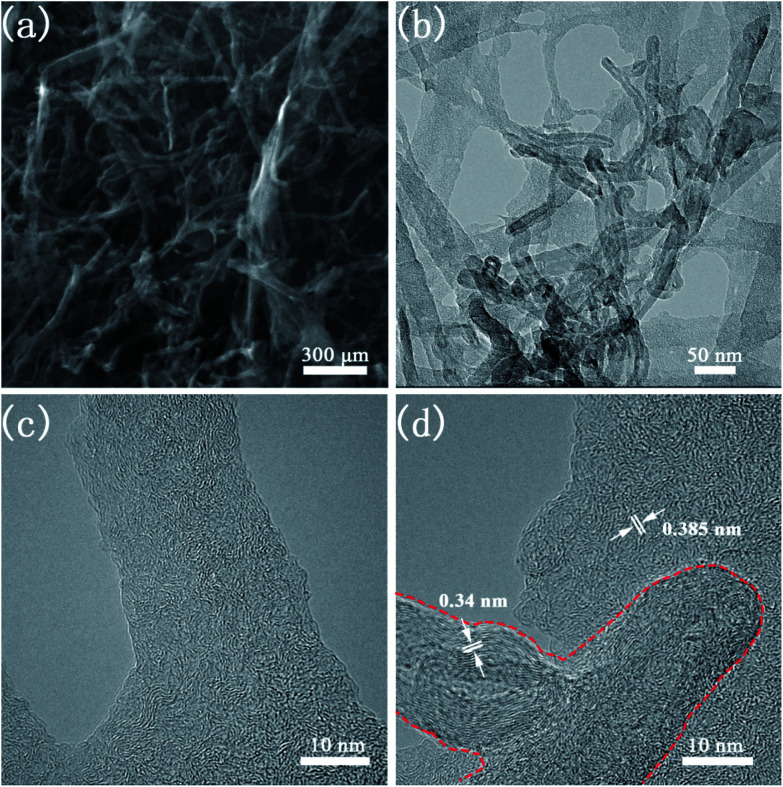
(a) SEM image of CNTs@N,O-CNFs; (b) TEM image of CNTs@N,O-CNFs; (c) HRTEM image of CNFs in CNTs@N,O-CNFs; (d) HRTEM image of MWCTs in CNTs@N,O-CNFs.

The morphology and microstructure of CNTs@N,O-CNFs were firstly investigated by scanning electron microscopy (SEM) and transmission electron microscopy (TEM). The SEM image in Fig. 1a shows the panoramic view of CNTs@N,O-CNFs, which revealed that the numerous ultrathin nanofibers were intertwined to form a porous network structure. It worth noting that a small quantity of CNTs was interspersed in the BC-derived nanofibers network, confirming the integration of two types of carbon materials. The TEM image in Fig. 1b gives direct evidence for the integration of CNFs and CNTs, in which some oriented CNTs are distributed in the CNFs network. It was found that most of CNTs exhibited a hollow structure. The HRTEM image in Fig. 1c shows that the CNFs consist of randomly oriented graphite-like regions and defective amorphous regions, which agrees well with the features of hard carbon. The HRTEM image in Fig. 1d clearly indicates that these CNTs are composed of layered graphitic carbon with a layer spacing of 0.34 nm (red dashed line area).^21^ The long nanofibers are connected to each other in all directions, ensuring continuous electron transport and shortening of the transmission path of Na ions. The incorporation of CNTs with CNFs may further increase the degree of graphitization of carbon materials, thereby the electrical conductivity of CNFs will be enhanced to reduce the charge transfer resistance of the carbon electrode. This assumption has been demonstrated by the following electrochemical impedance spectroscopic (EIS) measurements in Fig. S1.†

**Fig. 2:**
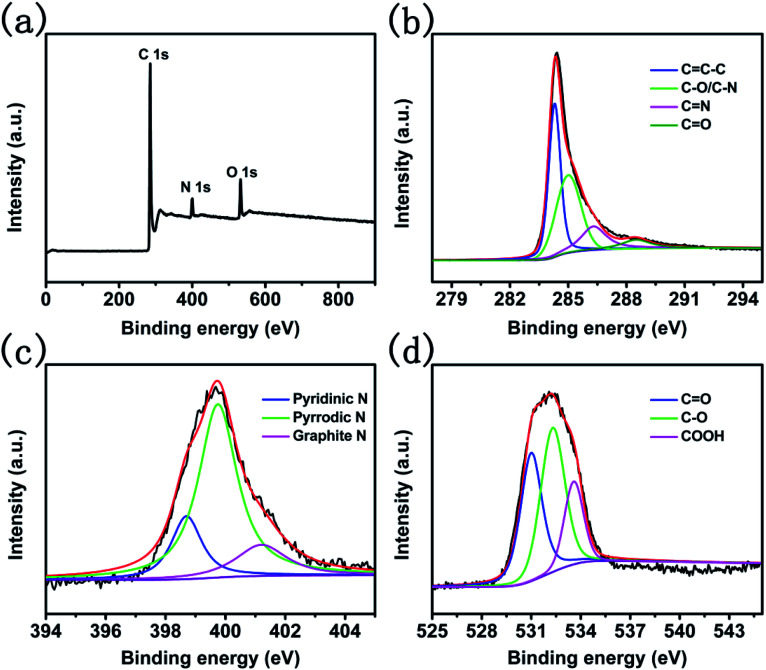
(a) The full XPS spectra of CNTs@N,O-CNFs and high resolution XPS spectra of (b) C 1s, (c) N 1s, and (d) O 1s.

To investigate the composition of CNTs@N,O-CNFs, energy dispersive X-ray (EDX) spectroscopic analysis was firstly performed. As shown in Fig. S2,† it can be seen that only C, N, and O elements were present in the EDX spectrum (Au came from the sputtered Au for SEM observation). The X-ray photoelectron spectroscopic (XPS) measurement was further performed to determine the relative contents of C, N, and O elements in CNTs@N,O-CNFs. The survey spectrum in Fig. 2a shows one predominant peak at 285.6 eV, and two medium peaks at 399.9 eV and 531.1 eV, which correspond to the characteristic peaks of C 1s, N 1s, and O 1s, respectively.^22^ The relative contents of C, N, and O was determined to be 86.06%, 5.56%, and 8.37%, respectively, which is basically consistent with the EDX result. The high-resolution C 1s spectrum in Fig. 2b possesses four types of peaks located at 284.8, 285.6, 287.1, and 289.36 eV, which could be ascribed to the C=C–C, C–N/C–O, C=N, and C=O bonds, respectively.^23^ The high resolution XPS N 1s spectrum shown in Fig. 2c can be divided into three types of peaks located at the binding energies of 398.7 eV, 399.7 eV, and 401.2 eV, which could be assigned to pyridinic-N, pyrrolic-N, and graphitic-N, respectively.^23^Fig. 2d shows the high-resolution XPS O 1s spectrum, in which three peaks centered at 532.0, 533.3, and 536.7 eV can be discerned, corresponding to C=O, C–O, and COOH bonds, respectively.^24^ The XPS result directly confirmed that the N and O elements have been doped into the carbon skeleton. This is because ethylenediamine itself possesses reducibility and has a high N-containing content of 46.67%. On the other hand, carbonized products from BC after oxidation possess O-containing functional groups, which can ensure that CNTs@CNFs react with ethylenediamine at a low temperature to provide N and O dual-doped carbon materials. More importantly, N-doping might increase the surface activity of the carbon material to improve the wettability between the electrode and the electrolyte. The most critical is that N and C atoms are covalently bonded, which could support the electrochemical reactivity and stability of the active materials.^25,26^

**Fig. 3:**
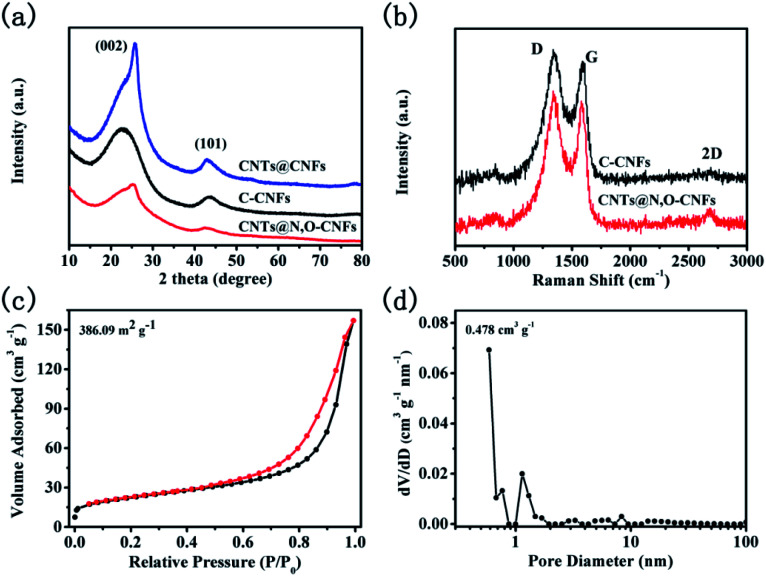
(a) XRD patterns of CNTs@N,O-CNFs, CNTs@CNFs, and C-CNFs, and (b) Raman spectra of CNTs@N,O-CNFs and C-CNFs; (c) nitrogen adsorption/desorption isotherms and (d) the corresponding pore-size distribution curve from the adsorption isotherms of the CNTs@N,O-CNFs composite.

The XRD patterns of C-CNFs, CNTs@N,O-CNFs, and CNTs@CNFs are shown in Fig. 3a. The XRD pattern of C-CNFs displayed two broad diffraction peaks for (002) and (101) centered at 23.07° and 44.01°, respectively,^27,28^ which is typical for the hard carbon. According to Bragg's formula, the interlayer *d*(002)-spacing was calculated to be 0.385 nm, which is beneficial for the de/intercalation of sodium ions between the layers.^29^ It can be noted that the (002) peaks shifted to a higher degree (from 23° to 25°) for the CNTs@N,O-CNFs and CNTs@CNFs due to the integration of CNTs with CNFs. The (002) peak for the CNTs@N,O-CNFs and CNTs@CNFs is centered at 25.18° and 25.69°, respectively. The extent of graphitization of CNTs@N,O-CNFs and C-CNFs was investigated by Raman spectroscopy (Fig. 3b). Two prominent peaks at ∼1350 cm^−1^ and ∼1600 cm^−1^ can be attributed to the disorder induced feature (D band) and the E_2g_ mode of graphite (G band),^30^ respectively. The intensity ratio of the G to D band (*I*_G_/*I*_D_) reflects the degree of graphitic ordering in the carbon materials.^31^ The *I*_G_/*I*_D_ ratio of CNTs@N,O-CNFs (0.62) is evidently larger than that of C-CNFs (0.546), which means that CNTs@N,O-CNFs has less defects and structural deformation after combination with highly ordered CNTs. In addition, the second characteristic peak of 2D appears at about 2700 cm^−1^, indicating that more stacked graphite-like layers are presented in CNTs@N,O-CNFs, which is an evidence of the high ordered degree of the graphitic layers as well. In order to clarify the specific surface area and pore-size distribution of CNTs@N,O-CNFs, N_2_ adsorption/desorption isotherm measurements were performed. As exhibited in Fig. 3c, a type-IV desorption isotherm with a type-H3 hysteresis loop appears in the range of *P*/*P*_0_ = 0.45–1.0. In addition, an upward trend of the isotherm at low relative pressure range (*P*/*P*_0_ = 0–0.45) was also observed, which confirms the presence of mesopores in carbon. The pore diameter distribution curve in Fig. 3d reveals that a large quantity of mesopores exists in CNTs@N,O-CNFs, whose pore size is mainly centered at 12.4 nm. The specific BET surface area was calculated to be 386.09 m^2^ g^−1^.

**Fig. 4:**
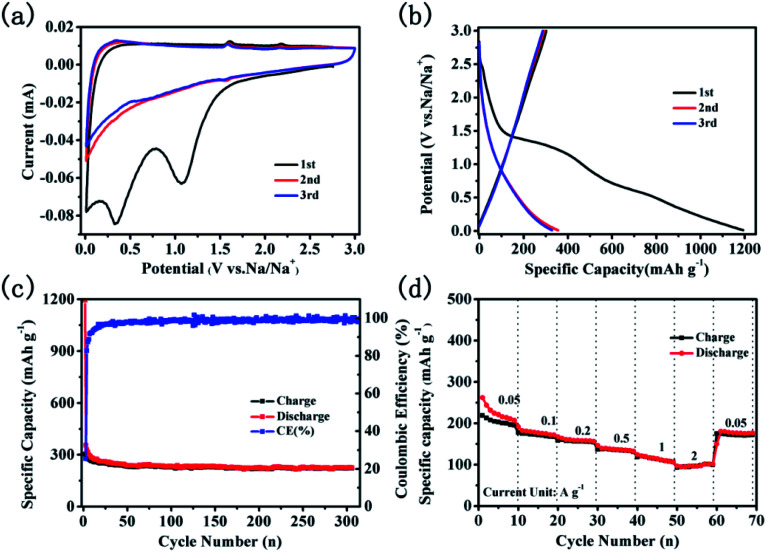
Electrochemical performance of the CNTs@N,O-CNFs electrode; (a) three initial CV curves at a scan rate of 0.1 mV s^−1^ in the potential range of 0.01–3 V; (b) voltage profiles for the 1^st^, 2^nd^, and 3^rd^ cycles at 50 mA g^−1^; (c) cycling performance for 300 cycles at 50 mA g^−1^; (d) rate performance at various current magnitudes in the range of 0.05 to 2 A g^−1^.

The electrochemical performance of CNTs@N,O-CNFs as the anode material was evaluated with Na metal as the counter electrode in the half cells. Fig. 4a displays the cyclic voltammogram (CV) curves for the first three cycles at a scan rate of 0.1 mV s^−1^ in the potential range of 0.01–3 V. Two irreversible cathode peaks at a potential of about 1.08 and 0.33 V appeared in the first scanning. The reduction peaks at 1.08 V can be assigned to the irreversible reaction of the electrolyte with the surface functional groups^32^ and the another peaks at 0.33 V may due to the decomposition of the electrolyte and the formation of the solid electrolyte interphase (SEI) film.^33,34^ On the basis of the previous report, the redox peaks at 0.01–0.3 V should correspond to the de/intercalation of sodium ions between the carbon layers.^35^ Another two pairs of weak peaks at about 1.53/2.18 V and 0.60/1.58 V in the redox process may be attributed to two pairs of redox reactions.^15^ The CV curves almost overlap in the subsequent cycles, suggesting the stability and reversibility of the de/intercalation process of sodium ions in CNTs@N,O-CNFs composite. The voltage profiles for the 1^st^, 2^nd^, and 3^rd^ cycles at a current density of 50 mA g^−1^ are shown in Fig. 4b. In the first cycle, the charge/discharge curves reveal that the charge/discharge specific capacity of the electrode is 302.9 and 1192.7 mA h g^−1^. The initial coulombic efficiency was calculated to be 25.4%. The large initial irreversible capacity of ∼890 mA h g^−1^ can be ascribed to the formation of the SEI layer at the surface of CNTs@N,O-CNFs, which is attributed to the reduction of the electrolyte and/or to irreversible sodium insertion into special positions in the vicinity of the carbon material.^36,37^Fig. 4c displays the long term cyclability of CNTs@N,O-CNFs at a current density of 50 mA g^−1^. Basically, the CNTs@N,O-CNFs electrode shows stable charge/discharge cycles; the coulombic efficiency can reach ∼100% after the first cycle. A stable reversible capacity of 225.8 mA h g^−1^ could be retained over 300 cycles. Notably, the CNTs@N,O-CNFs anode shows excellent rate performance. As shown in Fig. 4d, the reversible specific capacities at the current densities of 0.05, 0.1, 0.2, 0.5, and 1 A g^−1^ are 227.7, 181.8, 160.2, 139.5, and 117.2 mA h g^−1^, respectively. Even at a high rate of 2 A g^−1^, a reversible specific capacity of ∼98.2 mA h g^−1^ could still be delivered. Moreover, when the current density reversed to 50 mA g^−1^, the reversible capacity could recover to 174.3 mA h g^−1^. The superior rate capability of CNTs@N,O-CNFs may originate from the combination of CNTs with CNFs, which improved the graphitization degree and increased the electrical conductivity of the carbon materials. As demonstrated from the EIS spectrum in Fig. S1,† CNTs@N,O-CNFs reveals a smaller semicircle compared with the C-CNFs, indicating that CNTs@N,O-CNFs has lower charge transfer resistance than that of C-CNFs.

**Fig. 5:**
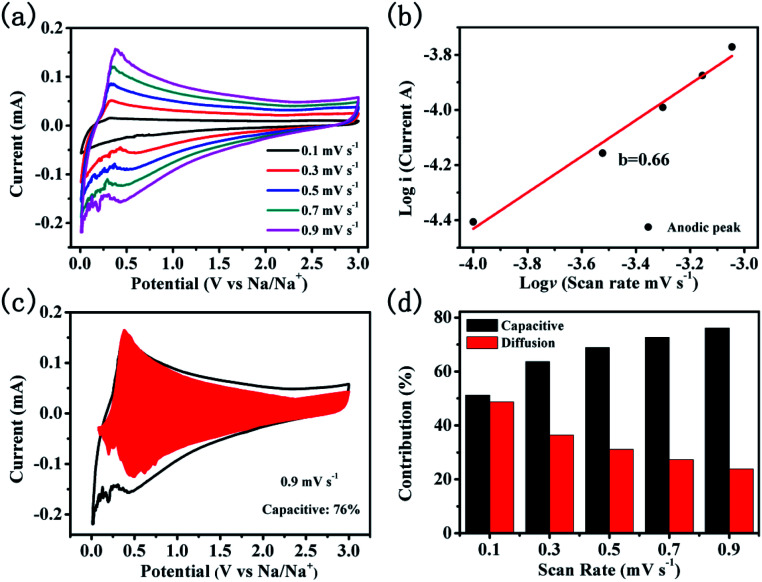
Quantitative capacitive analysis of sodium storage mechanism. (a) CV curves of the CNTs@N,O-CNFs electrode at various of scan rates; (b) relationship of the cathodic peak current and the scan rates for the CNTs@N,O-CNFs electrode; (c) capacitive contribution of the CNTs@N,O-CNFs; (d) charge contributions from the diffusion/capacitive-controlled process as a function of scan rate.

To reveal the sodium storage mechanism of CNTs@N,O-CNFs, kinetic analysis based on the CV curves was performed. Fig. 5a shows the CV curves of CNTs@N,O-CNFs at various scan rates from 0.1 mV s^−1^ to 0.9 mV s^−1^. The shape of the CV curves remains almost unchanged when the scan rate is increased. The peak current (*i*) and the scan rates (*ν*) from the CV curves can be quantitatively analyzed by the relationship: *i* = *aν*^*b*^, in which both *a* and *b* are adjustable parameters.^38^ The value of *b* is between 0.5 and 1, which can be determined by the slope of log(*i*) *versus* log(*ν*) plot. For the diffusion-controlled electrode reaction, *b* is close to 0.5, while for the surface capacitance-dominated process, *b* approaches 1.^39^ In general, the current response (*i*) is always derived from the combination of diffusion-controlled reaction and surface capacitive storage. Thus, the extent of capacitance contribution to the current can be quantitatively evaluated by the equation: *i*(*v*) = *k*_1_*v* + *k*_2_*v*^1/2^, where *k*_1_*v* represents the surface capacitance contribution and *k*_2_*v*^1/2^ corresponds to the current generated by the diffusion-controlled reaction.^40^ The constants *k*_1_ and *k*_2_ can be determined from the CV curves at various scan rates. As shown in the red area of Fig. 5c, 76% of the total capacity is defined as the capacitive contribution to the electrode at a scan rate of 0.9 mV s^−1^. According to the same method, the capacity contribution from the diffusion-controlled reaction and the surface-capacitive process as a function of the scan rate can be determined and the result is shown in Fig. 5d. As expected, the capacitive contribution gradually improves with an increase in the scan rate. The surface capacitance dominated kinetics of CNTs@N,O-CNFs that originates from the N and O double doping may account for the enhanced rate capability and cycling performance.

### 3.2 Positive electrode materials

**Fig. 6:**
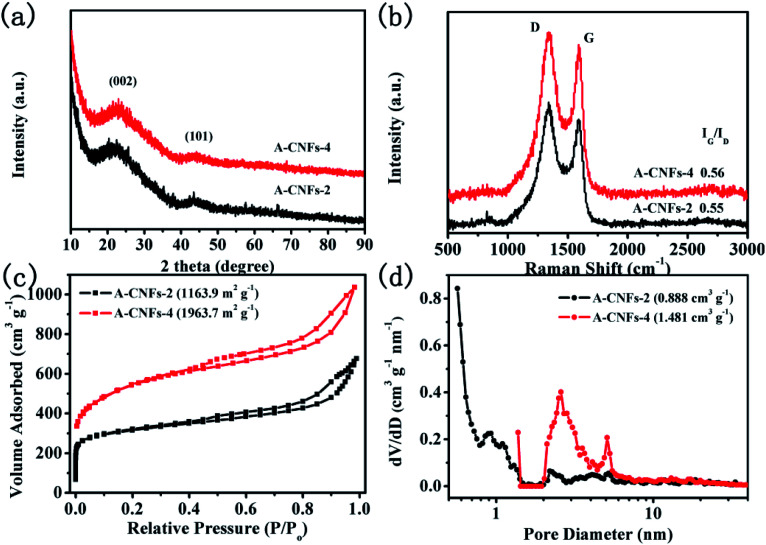
(a) XRD patterns and (b) Raman spectra of A-CNFs-2 and A-CNFs-4; (c) N_2_ adsorption/desorption isotherms and (d) pore diameter distribution of A-CNFs-2 and A-CNFs-4.

Considering that asymmetric supercapacitors with high energy and power density also depend on the electrochemical properties of positive electrode materials, it is essential to obtain positive electrode materials with high capacitive performance. Hence, the electrode material with a high specific surface area that can meet the capacitance reactions for rapid charge and discharge is required. In terms of the above considerations, CNFs was chemically activated by KOH to obtain porous CNFs so as to enhance the specific surface area. In addition, the porosity of CNFs is expected to be optimized. In order to optimize the activation process, different mass ratios of KOH were used to activate the carbon materials. The corresponding carbon materials are denoted as A-CNFs-2 (*W*_KOH_ : *W*_C-CNFs_ = 2 : 1) and A-CNFs-4 (*W*_KOH_ : *W*_C-CNFs_ = 4 : 1). SEM observation was firstly performed to investigate the effect of KOH activation on the morphology of CNFs. As shown in Fig. S3,† the 3D porous network structure of A-CNFs was maintained after activation at a temperature of 800 °C. Furthermore, there is no significant difference in the morphology and structure between A-CNFs-2 and A-CNFs-4. Fig. 6a shows the XRD patterns of A-CNFs-2 and A-CNFs-4. Both the activated CNFs display nearly identical XRD diffraction patterns. Two broad diffraction peaks for (002) and (101) centered at 23° and 43°, respectively, are presented in the pattern, which is typical of amorphous carbon. Raman spectroscopy was further performed to examine the extent of graphitization of CNFs after activation (Fig. 6b). Both A-CNFs show D bands (∼1350 cm^−1^) caused by the structural defects and G bands (∼1590 cm^−1^) caused by the first-order scattering of the E_2g_ mode observed for sp^2^-carbon domains.^41^ The intensity ratio of the G to D band (*I*_G_/*I*_D_) reflects the order degree of graphite in the carbon materials.^31^ The *I*_G_/*I*_D_ ratio of A-CNFs-2 and A-CNFs-4 was found to be 0.55 and 0.56, respectively, which is nearly consistent with that of C-CNFs (0.546). This result suggests that the degree of graphitization for CNFs does not change after chemical activation. In order to investigate the specific surface area and the pore size distribution of A-CNFs-2 and A-CNFs-4, BET measurements were performed. As exhibited in Fig. 6c and d, both A-CNFs showed type-IV H3 desorption isotherms with an upward trend at low pressure, in which the upward trend of A-CNFs-2 is more obvious than that of A-CNFs-4. Additionally, A-CNFs-2 and A-CNFs-4 reveal almost the same hysteresis loops in the relative pressure range of 0.45 to 1, which reveals the presence of micro/mesopores.^42^ The detailed parameters from the BET measurement are displayed in Table 1, which suggest that chemical activation by KOH at high temperature effectively increased the specific surface area and the pore volume.

**Table 1:** Physicochemical properties and average pore size of A-CNFs

	SSA^a^ (m^2^ g^−1^)	*V*_total_ b (cm^3^ g^−1^)	SSA_Micro_^c^ (m^2^ g^−1^)	*V*_Micro_ d (cm^3^ g^−1^)	*D*_average_ e (nm)
A-CNFs-2	1163.9	0.888	798.74	0.332	3.59
A-CNFs-4	1963.7	1.481	1212.57	0.525	3.26

a Specific surface area.

b Total volume.

c Specific pore surface area.

d Micropore volume.

e Average pore diameter.

**Fig. 7:**
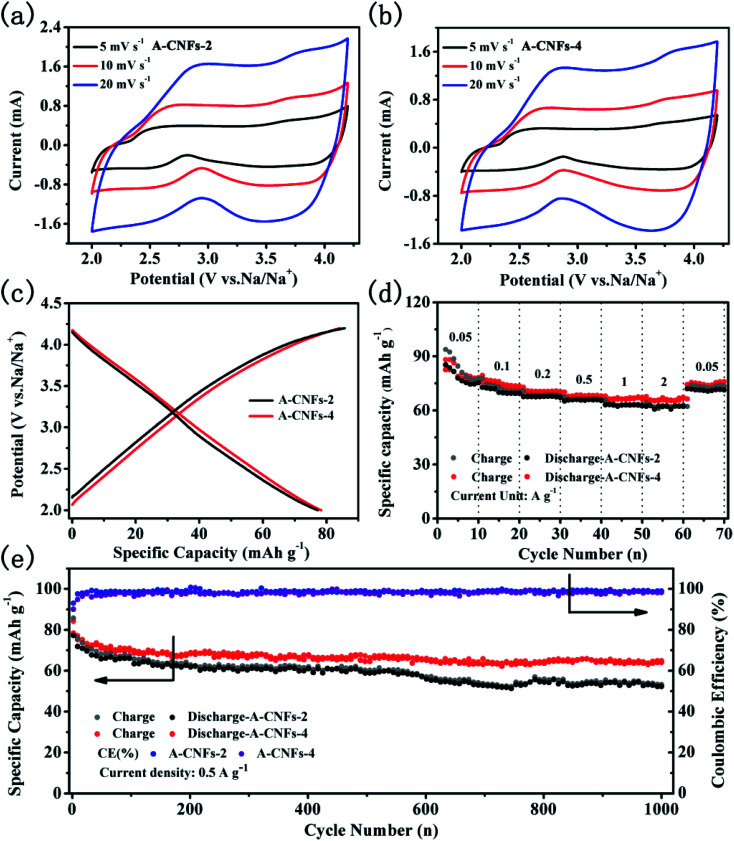
CV curves of (a) A-CNFs-2 and (b) A-CNFs-4 at various scan rates in the range of 5 to 20 mV s^−1^ in the potential range of 2–4.2 V; (c) voltage profiles of A-CNFs for the 1^st^ cycle at 50 mA g^−1^; (d) rate performance of A-CNFs at various current magnitudes in the range of 0.05 to 2 A g^−1^; (e) cycling performance of A-CNFs for 1000 cycles at 0.5 A g^−1^.

The electrochemical capacitive performance of both A-CNFs was examined by half-cell measurements with Na metal as the counter electrode. As shown in Fig. 7a and b, the CV curves of both A-CNFs-2 and A-CNFs-4 exhibit quasi-rectangular shapes, suggesting a typical double electrical layer capacitance (EDLC) behavior wrapped with pseudocapacitive humps.^43,44^ Accordingly, the voltage profiles of A-CNFs-2 and A-CNFs-4 in Fig. 7c appear as almost straight lines in the voltage range of 2.0–4.2 V.^45^ The charge specific capacity and discharge specific capacity of A-CNFs-2 and A-CNFs-4 are 85.7, 77.2 mA h g^−1^ and 84.2, 78.3 mA h g^−1^, respectively. The rate performance for A-CNFs is displayed in Fig. 7e. When the current density gradually increases from 0.05 to 0.1, 0.2, 0.5, 1, and 2 A g^−1^, the corresponding reversible specific capacity of A-CNFs-2 decreases from 82.21 to 72.65, 68.61, 66.28, 63.48, and 62.45 mA h g^−1^, respectively. In contrast, the reversible specific capacity of A-CNFs-4 decreases from 82.5 to 75.48, 70.89, 68.36, 66.81, and 66.05 mA h g^−1^, respectively. Fig. 7f presents the cycling stability of the positive electrodes over 1000 cycles at the current density of 0.05 A g^−1^. The retention rate of the discharge specific capacities over 1000 cycles for A-CNFs-2 and A-CNFs-4 are 62% and 78%, respectively. On the basis of electrochemical measurement results, the electrochemical capacitive performance of A-CNFs-4 is superior to that of A-CNFs-4, which can not only be seen from the value of specific capacity but also the capacity retention rate over long-term cycling.

### 3.3 Hybrid ion capacitor device

**Fig. 8:**
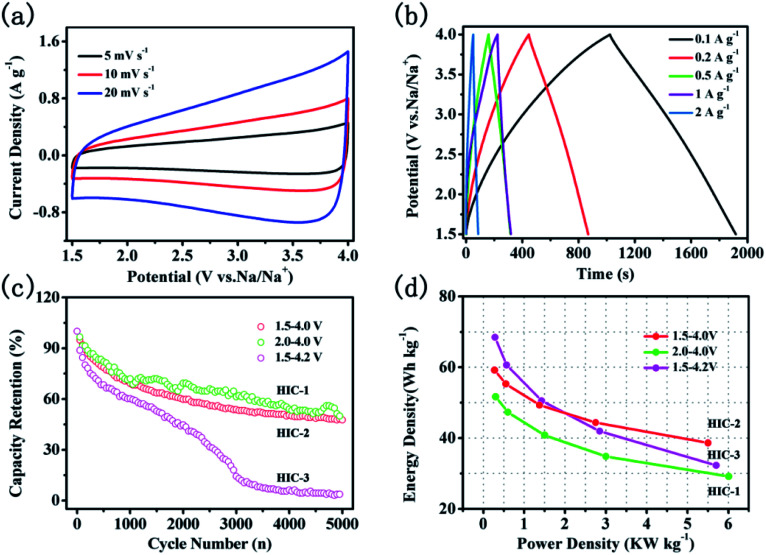
Electrochemical performance of CNTs@N,O-CNFs//A-CNFs-4; (a) CV curves at various scan rates in the range of 5 to 20 mV s^−1^ in the potential range of 2–4 V; (b) galvanostatic charge/discharge curves at various current densities from 0.1 to 2 A g^−1^; (c) the capacity retention over 5000 cycles at the current magnitude of 0.5 A g^−1^; (d) Ragone plots for various voltage ranges.

To examine the electrochemical capacitive performance of double carbon sodium ion capacitor, a hybrid sodium ion capacitor (HIC) was assembled using A-CNFs-4 as the positive electrode and CNTs@N,O-CNFs as the negative electrode. Before assembling the HIC, the negative electrode was charge-discharged for 5 cycles at a current density of 50 mA g^−1^ in the working potential window of 0.01–3 V. The mass ratio of the active substance in the negative and positive electrode is 1 : 2. According to the equation of *E* = ½*CV*^2^, the working voltage of HIC has a significant impact on the energy density of the full cell. To investigate the effects of the working voltage on the electrochemical performance of the assembled HIC, a variety of voltage ranges were studied such as, 2.0–4.0 V, 1.5–4.0 V, and 1.5–4.2 V, respectively. The assembled devices in the voltage range of 2.0–4.0 V, 1.5–4.0 V, and 1.5–4.2 V were named as HIC-1, HIC-2, and HIC-3, respectively. As shown in Fig. 8a, S4a and S4c,† all the CV curves of the HICs show quasi-rectangular shapes without obvious redox peaks at various voltage ranges, which revealed almost ideal capacitive behavior. As the scan rates increased from 5 to 20 mV s^−1^, the quasi-rectangular shapes of CV curves were retained without obvious deformation, indicating that the assembled HICs have good reversibility. The galvanostatic charge/discharge curves from the HICs (Fig. 8b, S4b and S4d†) at variety of current densities are approximately linear and symmetrical, confirming rapid response and excellent electrochemical reversibility.^10^ The calculated specific capacitance based on the total electrode active mass decreased from 31 to 20.2 F g^−1^ when the current densities increased from 0.1 to 2 A g^−1^. The decrease in the specific capacitance is mainly due to the large voltage drops, and poor contact between the electrolyte and electrode materials at high current densities.^46^

Fig. 8c shows the capacity retention rate of HICs at a current density of 0.5 A g^−1^ after 5000 cycles. When the voltage range is set as 1.5–4.0 V, the capacity retention rate of HIC-2 is 48.6%, which is close to that of HIC-1 (50.7%) but is much higher than that of HIC-3 (14.6%). The large capacity loss of HIC-3 could be due to decomposition of the electrolyte at high voltage near the end of the charge.^47,48^ The Ragone plot of the present HIC assembled by the CNTs@N,O-CNFs and the A-CNFs-4 in different voltage ranges is shown in Fig. 8d. As can be seen, the energy density of HIC-1 is always lower than that of HIC-2 and the HIC-3 since a narrower voltage range directly leads to a lower energy density, according to the formula of *E* = ½*CV*^2^. Meanwhile, the energy density of HIC-3 is higher than that of HIC-2 at a low power density. When the power density increased to ∼2.75 kW kg^−1^, however, the energy density of HIC-2 as calculated to be ∼44.4 W h kg^−1^, which is slightly higher than that of HIC-3 (∼41.2 W h kg^−1^). Moreover, even at a power density of 5.5 kW kg^−1^, HIC-2 retained a high energy density of 38.7 W h kg^−1^, suggesting a better cycle performance than that of the HIC-3. It is probably due to the fact that compared to HIC-2, the wider voltage window of HIC-3 intensified the over-charging/discharging of the device under high current density, which might lead to structural damage of the electrode material and therefore, the cycling stability of the device was affected. The lower capacity retention rate of HIC-3 in Fig. 8c also confirms the above deduction. It is significant to compare the electrochemical performance of the present CNTs@N,O-CNFs//A-CNF device with the previously reported promising hybrid ion capacitor systems. It can be seen from Table S1† that the energy density and power density of HIC-2 is comparable to previously reported asymmetric devices, such as CS-800//CS-800-6,^7^ Na-TNT//AC,^49^ NVP@AC//NVP@AC,^50^ Nb_2_O_5_//AC,^51^ MWTOG//AC,^52^ and V_2_O_5_@CNT//AC.^53^

## 4. Conclusions

In summary, a hybrid dual carbon sodium ion capacitor was assembled by bacterial cellulose derived carbon nanofibers as both the anode and the cathode. The incorporation of CNTs increased the degree of graphitization and reduced the charge transfer resistance of CNFs, which, combined with the N and O heteroatoms, double the doping effectively, and improved the rate capability and cycling performance of the carbon anode. Chemical activation with KOH optimized the porosity of CNFs to obtain a high specific capacity carbon cathode. Meanwhile, with optimized voltage windows, the assembled double carbon sodium ion capacitor can offer a high energy density of 59.2 W h kg^−1^ at a power density of 276 W kg^−1^. When the power density was increased to 5500 W kg^−1^, an energy density of 38.7 W h kg^−1^ could still be maintained. The capacity retention of the device was 48.6% at a current density of 0.5 A g^−1^ in the voltage range of 1.5–4.0 V after 5000 cycles. Such a “double carbon” configuration for the device and electrode structure design reduced the mismatch of electrode kinetics between the anode and the cathode, which led to a high energy density and high power density. Most importantly, the electrode materials come from eco-friendly biomaterials, which, combined with the abundance of sodium resource on earth, makes the assembled sodium ion capacitor a potential electrical energy storage device.

## Conflicts of interest

The authors declare no conflict of interest.

## Supplementary Material

RA-010-C9RA10225F-s001
