# Supplementary material for: Bacterial cellulose-derived carbon nanofibers as both anode and cathode for hybrid sodium ion capacitor
Source: RSC Adv. 2020 Feb 24;10(13):7780–90. doi: 10.1039/c9ra10225f (PMC9049867; doi:10.1039/c9ra10225f)
Supplement: RA-010-C9RA10225F-s001 [file RA-010-C9RA10225F-s001.pdf]

Electronic Supplementary Material (ESI) for RSC Advances.
This journal is © The Royal Society of Chemistry 2020

Supporting Information

Bacterial Cellulose Derived Carbon Nanofibers as both Anode and Cathode for Hybrid Sodium Ion Capacitor

Jiaxin Xu, Zhanying Liu, Fang Zhang*, Jie Tao, Laifa Shen, Xiaogang Zhang*

Jiangsu Key Laboratory of Materials and Technology for Energy Conversion, College of Material Science and Engineering, Nanjing University of Aeronautics and Astronautics, Nanjing 210016, People's Republic of China

*Corresponding authors. E–mail: zhangfang@nuaa.edu.cn

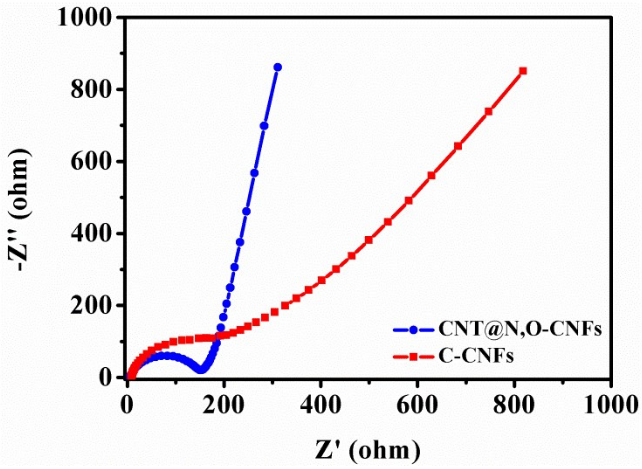

Figure S1. Nyquist plots of the CNT@N,O–CNFs and C-CNFs electrodes

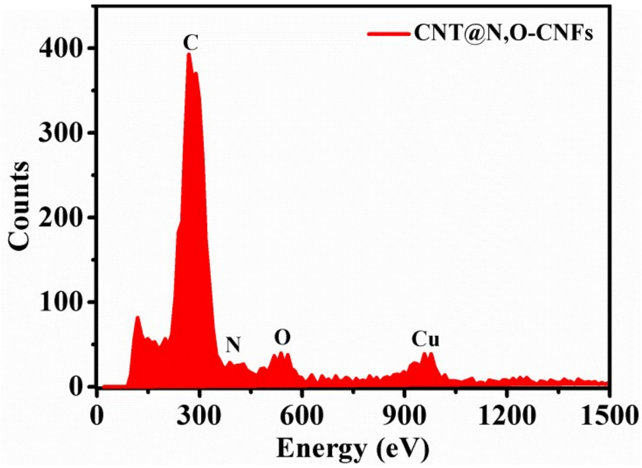

Figure S2. Energy dispersive X-ray spectroscopy analysis of CNT@N,O–CNFs

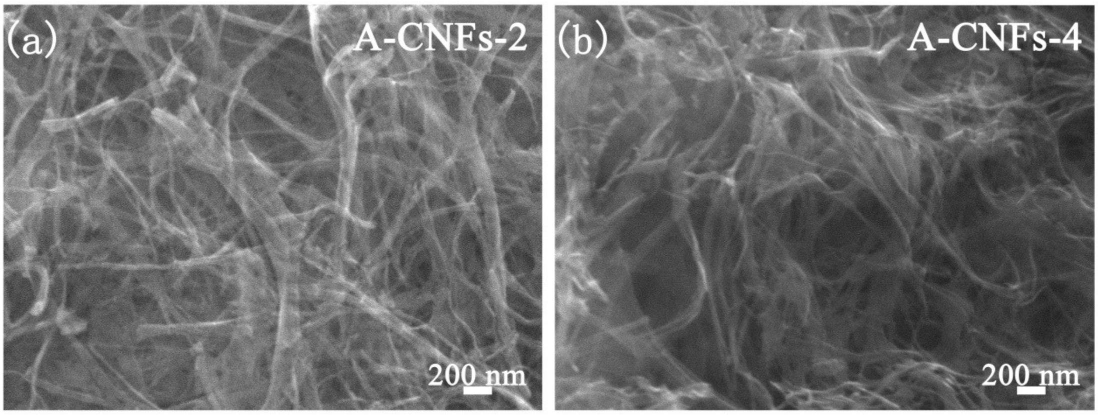

Figure S3. SEM images of (a) A-CNFs-2, (b) A-CNFs-4

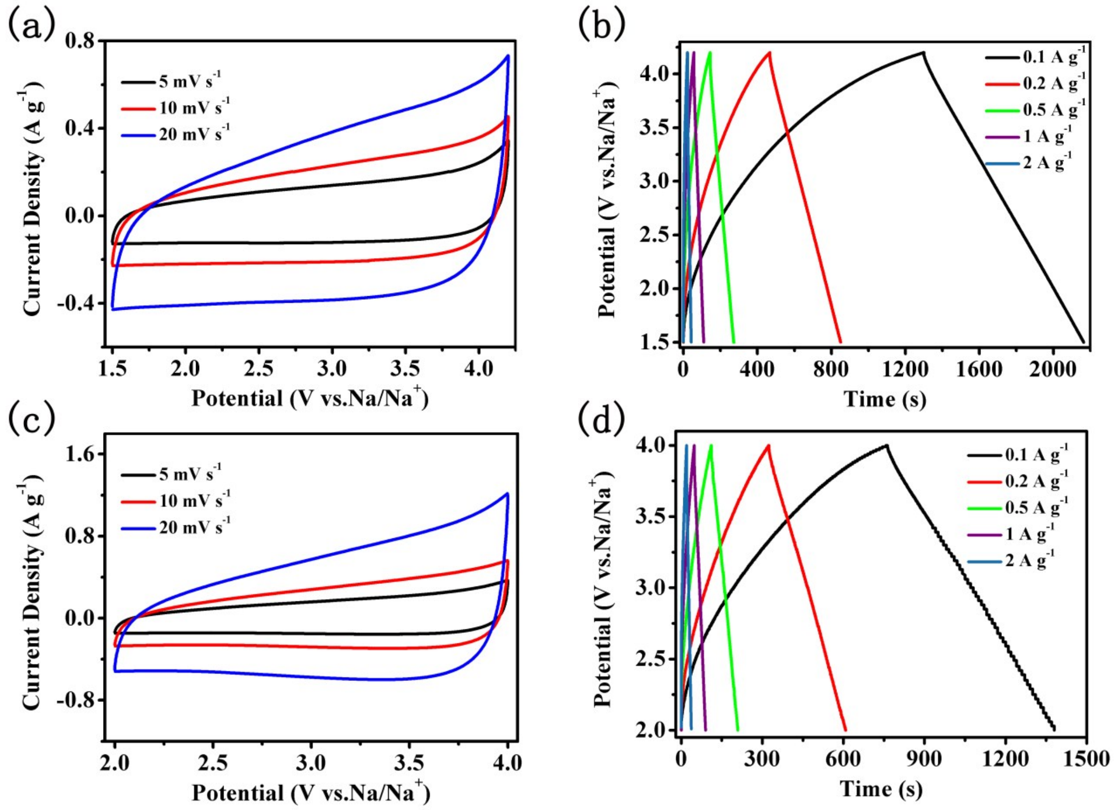

Figure S4. CV curves at various scan rates of 5 to 20 mV s^{-1} in a potential range of (a) 1.5-4.2 V and (c) 2.0-4.0 V; Galvanostatic charge/discharge curves with various current densities from 0.1 to 2 A g^{-1} in a potential range of (b) 1.5-4.2 V and (d) 2.0-4.0V;

Table S1. Comparison of the electrochemical performance of full-cell HICs

System (anode//cathode)	Energy density ($Wh\ kg^{-1}$)	Power density ($W\ kg^{-1}$)	Capacity retention (%)/cycle number/current density ($A\ g^{-1}$)	Ref.
**CNTs@N,O-CNFs//A-CNFs**	59.2@275 $W\ kg^{-1}$	5500@38.7 $Wh\ kg^{-1}$	48.6%/5000/0.5	This work
**CS-800//CS-800-6**	52.2@300 $W\ kg^{-1}$	3000@15.8 $Wh\ kg^{-1}$	85.7%/2000/1	1
**S-N/CNTs//AC**	116.4@200 $W\ kg^{-1}$	20000@48.2 $Wh\ kg^{-1}$	81%/3000/2	2
**Na-TNT//AC**	34@120 $W\ kg^{-1}$	889@13 $Wh\ kg^{-1}$	80%/1000/0.25	3
**NVP@AC//NVP@AC**	26@270W kg^{-1}	5424@15 $Wh\ kg^{-1}$	64.5%/10000/1.17	4
**Nb_2O_5//AC**	43.2@160 $W\ kg^{-1}$	5760@24 $Wh\ kg^{-1}$	80%/3000/1.28	5
**MWTOG//AC**	64.2@56 $W\ kg^{-1}$	1357@25.8 $Wh\ kg^{-1}$	90%/10000/3.35	6
**V_2O_5@CNT//AC**	38@140 $W\ kg^{-1}$	5000@7.5 $Wh\ kg^{-1}$	80%/900/60 C	7

References

1. S. Wang, R. Wang, Y. Zhang, D. Jin and L. Zhang, *J. Power Sources*, 2018, **379**, 33-40.
2. K. Liao, H. Wang, L. Wang, D. Xu, M. Wu, R. Wang, B. He, Y. Gong and X. Hu, *Nanoscale. Adv*, 2019, **1**, 746-756.
3. J. Yin, L. Qi and H. Wang, *Acs Appl. Mater. Interfaces*, 2012, **4**, 2762-2768.
4. Z. Jian, V. Raju, Z. Li, Z. Xing, Y. S. Hu and X. Ji, *Adv. Funct. Mater.*, 2015, **25**, 5778-5785.
5. H. Li, Y. Zhu, S. Dong, L. Shen, Z. Chen, X. Zhang and G. Yu, *Chem. Mater.*, 2016, **28**, 5753-5760.
6. Z. Le, F. Liu, P. Nie, X. Li, X. Liu, Z. Bian, G. Chen, H. B. Wu and Y. Lu, *ACS nano*, 2017, **11**, 2952-2960.
7. Z. Chen, V. Augustyn, X. Jia, Q. Xiao, B. Dunn and Y. Lu, *ACS nano*, 2012, **6**, 4319-4327.
